# Fresh Beef and Lamb Consumption in Relation to Nutrient Intakes and Markers of Nutrition and Health Status among the Population Aged 5–90 Years in Ireland

**DOI:** 10.3390/nu15020313

**Published:** 2023-01-08

**Authors:** Laura Kehoe, Emma O’Sullivan, Chris Cocking, Breige A. McNulty, Anne P. Nugent, Kevin D. Cashman, Albert Flynn, Janette Walton

**Affiliations:** 1 Department of Biological Sciences, Munster Technological University, T12 P928 Cork, Ireland; 2 School of Food and Nutritional Sciences, University College Cork, T12 CY82 Cork, Ireland ,; 3 UCD Institute of Food and Health, University College Dublin, D04 V1W8 Dublin, Ireland; 4 School of Biological Sciences, Institute for Global Food Security, Queen’s University Belfast, Belfast BT9 5DL, UK

**Keywords:** fresh beef, fresh lamb, consumption, nutrient intakes, nutritional status

## Abstract

The dietary role of meat is under scrutiny for health and environmental reasons, yet a growing body of evidence proposes that advice to limit red meat consumption is unnecessarily restrictive. The aim of this study was to investigate the role of ‘fresh beef and lamb’ in the diet of the population (5–90 years) in Ireland and its association with markers of nutrition and health status. Analyses are based on data from three nationally representative dietary surveys in the Republic of Ireland. Dietary intake data were estimated using food records, and nutrient intakes were estimated based on UK and Irish food composition tables. Biochemical samples were collected and analysed using standard procedures. ‘Fresh beef and lamb’ (defined as beef/lamb that had not undergone any preserving process other than chilling/freezing/quick-freezing) was consumed by 68–84% of the population and intakes ranged from 19 to 43 g/d across age groups. It made important contributions to intakes of protein, monounsaturated fat, vitamins D, B12, niacin, iron and zinc while also contributing relatively small proportions of total fat, saturated fat and salt. Higher consumption of ‘fresh beef and lamb’ was associated with higher intakes of protein, niacin, vitamins B6, B12, zinc and potassium (but also total fat) and lower intakes of carbohydrate and total sugars (but also dietary fibre). In adults, older adults and WCBA, higher consumption of ‘fresh beef and lamb’ was not associated with increased risk factors of cardio-metabolic diseases nor was it associated with better or poorer nutritional status for vitamins D, B12 or iron. This study adds to the evidence base on the contribution of ‘fresh beef and lamb’ in the diet and may be useful to policymakers updating guidance for healthy diets from sustainable food systems.

## 1. Introduction

The role of meat in the diet is currently under scrutiny for both health and environmental reasons, amplified by the recent report of the EAT-Lancet commission recommending an extreme reduction in the consumption of processed and red meat as part of a healthy diet from sustainable food systems [1]. Food-based dietary guidelines (FBDG) in the developed world recommend consuming lean meat in some form due to its contribution to the intake of key nutrients such as protein, iron, zinc and vitamin B12, but to consume little if any processed meat and to limit the intake of red meat [2]. Furthermore, the World Cancer Research Fund (WCRF) recommends consuming < 500 g of red meat per week and very little if any processed meat, while the UK Scientific Advisory Committee on Nutrition (SACN) recommends limiting red and processed meat consumption to <70 g per day [3,4]. These limits on processed and red meat are based on findings from epidemiological studies which have suggested associations between red and processed meat consumption and an increased risk of non-communicable diseases including cardiovascular disease, type 2 diabetes mellitus and certain cancers [5,6,7,8,9].

However, a growing body of evidence proposes that dietary advice to limit red meat for health benefits is unnecessarily restrictive, with some literature suggesting that the causal relationships between red meat and mortality are not supported by the evidence and that the negative health outcomes previously linked to red meat consumption are associated with the wider dietary patterns associated with being a red meat consumer [10,11,12,13]. Furthermore, recent randomized controlled trials have shown no benefit of choosing white meat over red meat for reducing CVD risk based on lipid and lipoprotein effects and have also shown that the benefits of a healthy, low saturated fat, Mediterranean-style diet were not attenuated by the inclusion of smaller to moderate amounts (≤71 g/d) of lean beef [14,15]. Subsequently, many researchers are now emphasizing the importance of distinguishing between unprocessed red meats such as beef, veal, pork and lamb and processed meats such as bacon, sausages, bologna and salami [11,16,17,18] due to processed meats being a greater source of saturated fat and sodium in the diet than fresh unprocessed red meat [18]. 

Globally, national dietary surveys report that fresh red meat including beef and lamb (and their dishes) make important contributions to intakes of protein, vitamin B12, vitamin D, iron, zinc and selenium [19,20,21,22,23,24]. A recent study in the UK has shown that females (a population group who already have some of the lowest red meat intakes) with intakes of red meat < 40 g/day were more likely to have intakes of iron, zinc, vitamin B12 and potassium below the lower reference nutrient intake (LRNI) than those with intakes of >40 g/d [25]. Furthermore, studies of adolescent and adult groups have shown no difference in markers of health and cardio-metabolic diseases between those consuming up to 70 g/d or ≥6 servings/week of fresh red meat compared to those consuming smaller amounts/less frequently [26,27]. With the European Commission FOOD 2030 report on nutrition highlighting the development of FBDG for healthy and sustainable diets as an enabler of change [28] and countries globally incorporating environmental sustainability and sociocultural factors in their updated FBDG, it is necessary to have baseline information on the current contributions of food groups including red meat to the diets of population groups. Therefore, the aims of the current study are to investigate the role of ‘fresh beef and lamb’ in the diets of the population aged 5–90 years in Ireland including vulnerable groups such as children, women of childbearing age (WCBA) and older adults, and the association of ‘fresh beef and lamb’ with markers of nutrition and health status in adult population groups.

## 2. Materials and Methods

### 2.1. Study Sample 

Analyses for the present study are based on food consumption data for children, teenagers and adults in the Republic of Ireland (ROI) available through three nationally representative dietary surveys: the *National Children’s Food Survey* (NCFS) (5–12 years) (2003-04), the *National Teens’ Food Survey* (NTFS) (13–17 years) (2005-06) and the *National Adult Nutrition Survey* (NANS) (18–90 years) (2008-10) (www.iuna.net [accessed on 1 August 2022]). All studies were conducted according to the guidelines laid down in the Declaration of Helsinki and ethical approval was obtained from St James’ Hospital and Federated Dublin Voluntary Hospitals Joint Research Ethics Committee for the NCFS and the University College Cork Clinical Research Ethics Committee of the Cork Teaching Hospitals for the NTFS and the NANS. Written informed consent was obtained from the participants themselves for those aged ≥18 years and from the participants and their parents/guardians for those aged ≤17 years. 

### 2.2. Sampling and Recruitment

A sample of 594 children aged 5–12 years (boys: 293, girls: 301) and 441 teenagers aged 13–17 years (boys: 224, girls: 217) were selected from databases of primary and secondary schools provided by the Department of Education and Science for the NCFS and NTFS, respectively. For the NANS, a sample of 1500 adults (men: 740, women: 760), who were free-living and not pregnant or breastfeeding, were randomly selected from a database of names and addresses held by Data Ireland (A Post). Each survey was shown to be representative of the intended population group with respect to age group, sex, social class and geographical location when compared to the most recent census at the time of each survey [29,30]. The response rates for the NCFS, NTFS and NANS were 66%, 63% and 60%, respectively. 

### 2.3. Dietary Intake Assessment

Food and beverage consumption data were collected at brand level using a 7-day weighed food record for the NCFS, a 7-day semi-weighed food record for the NTFS and a 4-day semi-weighed food record for the NANS. A hierarchal method was used to quantify the amount of each food/beverage consumed and included direct weighing of the food by participants, weights provided on product labels or from manufacturers, use of a photographic food atlas [31], standard food portion sizes [32], household measures and estimated quantities (for a small number of foods based on participants previous habits). Nutrient intakes were estimated using WISP© (Tinuviel Software, Anglesey, UK), which contained food composition data from McCance and Widdowson’s *The Composition of Foods* sixth [33] and fifth [34] editions plus all nine supplemental volumes [35,36,37,38,39,40,41,42,43]. During each survey, modifications were made to the food composition database to include recipes of composite dishes, food supplements, fortified foods, generic Irish foods that were commonly consumed and to update values for total fat, saturated fat, monounsaturated fat (MUFA), polyunsaturated fat (PUFA), free sugar, sodium and vitamin D, the details of which are outlined in detail elsewhere [44,45,46,47]. To facilitate dietary intake assessment, participants were asked to provide the packaging labels of all food and beverages consumed.

### 2.4. Biomarkers of Nutritional and Health Status

In the NANS only, participants were also asked to provide a blood and urine sample, of which 76% of participants (*n* 1138) provided a blood sample (79% fasting) and 75% provided a morning first void urine sample (*n* 1121). Serum total cholesterol (mmol/L), serum TAG (mmol/L), serum direct HDL cholesterol (mmol/L), serum 25-hydroxyvitamin D (nmol/L), serum vitamin B12 (pmol/L), haemoglobin (g/dL) serum ferritin (ng/mL), urinary sodium (mmol/L), and urinary potassium (mmol/L) were measured using standard methodologies, as described in detail elsewhere [47,48,49,50]. LDL-cholesterol levels (mmol/L) were calculated as (Total cholesterol/HDL cholesterol) −(TAG/2·2). Urinary molar sodium potassium ratio (Na:K) was calculated for each participant as Na (mmol/L)/K(mmol/L), with a value of 0.6 being subtracted from each individual value to correct for timing bias associated with circadian rhythms [51]. Systolic and diastolic blood pressure (BP) were measured using a blood pressure monitor (OMRON M6 Comfort) by a trained researcher in the participant’s own home. The reading was taken in triplicate from the right arm, where possible, with five-minute intervals between each measurement by standard procedures. 

### 2.5. Defining ‘Fresh Beef and Lamb’

In the present analysis, ‘fresh beef and lamb’ included any beef or lamb that had not undergone any preserving process other than chilling, freezing or quick freezing and included beef or lamb which was vacuum wrapped or wrapped in a controlled atmosphere. Beef and lamb that had been treated with any preservatives (other than salt) was excluded from these analyses. Intakes of ‘fresh beef and lamb’ were estimated from discrete cuts and also from composite dishes (following disaggregation of the non-meat components, e.g., potatoes, pasta, vegetables, sauces and oils). Previous analysis has shown that failure to disaggregate composite foods substantially overestimates meat intakes by approximately 74% for beef and 51% for lamb [52]. 

### 2.6. Estimation of ‘Fresh Beef and Lamb’ Intake and Contribution to Energy and Nutrient Intakes

The mean daily intake (MDI) (g/d) of ‘fresh beef and lamb’ was calculated for individuals by summing their total intake of ‘fresh beef and lamb’ over the survey period and dividing by the number of survey days (NCFS and NTFS: 7 days, NANS: 4 days) for all population groups of interest (children, teenagers, adults, older adults, WCBA). Consumers were defined as those who consumed any amount of ‘fresh beef or lamb’ on any day during the survey period. The contribution of ‘fresh beef and lamb’ to intake of energy and selected nutrients was estimated including the non-meat components of composite dishes using the mean proportion method for ‘fresh beef and lamb’ consumers only. This method provides information about the sources that are contributing to the nutrient intake ‘per person’ and is the preferred method when determining important food sources of a nutrient for individuals in the population group as opposed to investigating the sources of a nutrient within the food supply [53]. 

### 2.7. Association of ‘Fresh Beef and Lamb’ Consumption with Nutrient Intakes, Biochemical Markers of Nutritional Status and Blood Pressure Measurements in Consumers Only

To identify any associations between ‘fresh beef and lamb’ consumption and energy and nutrient intakes (all population groups) or markers of nutrition and health status (for adults only), each population group was split into three equal tertiles (groups) based on their MDI of ‘fresh beef and lamb’ (stratified by sex and age group); non/low, medium and high consumers of ‘fresh beef and lamb’. Mean intakes of energy and nutrients (energy adjusted, excluding nutritional supplements), biochemical markers and systolic and diastolic BP were then compared between the non/low and high consumer groups. The proportion of the population with systolic BP ≥ 140 mmHg [54], diastolic BP ≥ 90 mmHg [54] serum total cholesterol > 5.2 mmol/L [55], serum 25-hydroxyvitamin D < 50 nmol/L [56], serum vitamin B12 < 148 pmol/L [57,58], serum ferritin < 15µg/L [3,59,60], haemoglobin < 13 g/dL (men) and <12 g/dL (women) [3] and urinary Na:K ratio > 1 [61] were also compared between the non/low and high consumer groups. To remove nutritional supplement use as a potential confounder, those using nutritional supplements containing vitamin D, vitamin B12 or iron were excluded from analyses on the association of ‘fresh beef and lamb’ consumption with nutritional status for these micronutrients.

### 2.8. Statistical Analyses

Statistical analysis was carried out using SPSS© for Windows™ Version 26.0. Differences in intakes of ‘fresh beef and lamb’, ‘beef’, ‘lamb’ and nutrient intakes between sexes, age groups and between consumer groups were assessed using independent sample t-tests for normally distributed data or for large sample sizes [62] and Mann–Whitney U tests for non-normal data. Differences in the proportion of consumers of ‘fresh beef and lamb’, ‘beef’ or ‘lamb’ and the proportion of the population with biochemical markers of nutritional status values outside of generally accepted cut-offs indicating high or low/deficient status were assessed using Chi-square tests. To minimise type 1 errors (as a result of multiple testing), the Bonferroni adjustment was used by dividing the alpha level (0.05) by the number of comparisons with intakes considered to be significantly different from each other if *p* < 0.001.

## 3. Results

### 3.1. Consumption of ‘Fresh Beef and Lamb’ 

‘Fresh beef and lamb’ was consumed by 84% of children (5–12 years) and teenagers (13–17 years), 76% of adults (18–64 years), 73% of older adults (65–90 years) and 68% of WCBA (Table 1). Beef was more commonly consumed than lamb for all age groups (children: 79 vs. 19%, teenagers: 83 vs. 20%, adults: 70 vs. 16%, older adults: 59 vs. 30% and WCBA: 63 vs. 11%). The MDI of ‘fresh beef and lamb’ for those aged 5–12 years was 19.2 g (beef: 16.6 g, lamb: 2.6 g) and for those aged 13–17 years was 32.8 g (beef: 28.3 g, lamb: 4.5 g) (Table 1). The MDI of ‘fresh beef and lamb’ was 42.7 g (beef: 36.3 g, lamb: 6.4 g) for those aged 18–64 years, 40.8 g (beef: 28.2 g, lamb: 12.6 g) for those aged 65–90 years and 27.0 g (beef: 23.7 g, lamb: 3.3 g) for WCBA. There were no differences in the MDI of ‘fresh beef and lamb’, ‘beef’ or ‘lamb’ between boys/men and girls/women for children or older adults (Table 1). However, compared to girls, teenage boys had a higher MDI of ‘fresh beef and lamb’ (40.2 vs. 25.1 g) and ‘beef’ (34.9 vs. 21.4 g), while there was no difference in the MDI of ‘lamb’. Similarly, compared to women aged 18–64 y, men had a higher MDI of ‘fresh beef and lamb’ (55.7 vs. 29.7 g), ‘beef’ (47.1 vs. 25.6 g) and ‘lamb’ (8.6 vs. 4.1 g). 

### 3.2. Contribution of ‘Fresh Beef and Lamb’ to Energy and Nutrient Intakes

In consumers of ‘fresh beef and lamb’, this food group contributed 5% of the MDI of energy in children and 7% in teenagers (Table 2). Relative to its contribution to energy intake for these population groups, ‘fresh beef and lamb’ contributed greater proportions of protein (12–15%) and MUFA (9–11%), similar proportions of total fat (7–9%), saturated fat (7–10%), PUFA (4–5%) and salt (6–7%) and smaller proportions of carbohydrate (2%), dietary fibre (2–3%), total sugars (1–2%) and free sugars (<1%). Relative to its contribution to energy intake, ‘fresh beef and lamb’ also contributed greater proportions of zinc (18–24%), vitamin B12 (15–22%), vitamin D (12–16%) and niacin (9–13%) and similar proportions of vitamin A (7%), iron (7–10%), vitamin B6 (6–9%), potassium (6–8%), vitamin K (4–6%) and total folate (3–4%). 

For ‘fresh beef and lamb’ consumers, this food group contributed 8% of the MDI of energy for adults and 10% for older adults, respectively (Table 2). Relative to its contribution to energy intake for these population groups, ‘fresh beef and lamb’ contributed greater proportions of protein (19–20%), total fat (12–14%), saturated fat (13–16%) and MUFA (16%) similar proportions of salt (8–9%) and PUFA (5–8%) and smaller proportions of carbohydrate (2%), dietary fibre (3%), total sugars (2%) and free sugars (≤1%). ‘Fresh beef and lamb’ also contributed greater proportions of vitamin B12 (30%), zinc (27%), niacin (15–18%), vitamin D (10–14%) and iron (12–13%) and similar proportions of vitamin B6 (11–12%), vitamin A (8–10%), vitamin K (5–6%), potassium (5–6%) and total folate (4%).

For ‘fresh beef and lamb’ consumers, this food group contributed 7% of the MDI of energy for WCBA (Table 2). Relative to its contribution to energy intake, ‘fresh beef and lamb’ contributed greater proportions of protein (17%), saturated fat (11%) and MUFA (11%), similar proportions of total fat (10%), salt (9%) and PUFA (5%) and smaller proportions of carbohydrate (2%), dietary fibre (3%), total sugars (2%) and free sugars (1%). ‘Fresh beef and lamb’ also contributed greater proportions of vitamin B12 (28%), zinc (25%), niacin (14%) and vitamin D (13%) and similar proportions of iron (11%), vitamin B6 (10%), vitamin A (7%), vitamin K (5%), potassium (5%) and total folate (4%).

### 3.3. Nutrient Intakes in Non/Low and High Consumers of ‘Fresh Beef and Lamb’

The MDI of energy was higher among high consumers of ‘fresh beef and lamb’ compared to non/low consumers for teenagers (2158 vs. 1861 kcal), adults (2106 vs. 1919 kcal) and WCBA (1843 vs. 1634 kcal) while there was no difference in the intake of energy between high and non/low consumer groups for children or older adults (Table 3). The MDI of protein was higher among high consumers compared to non/low consumers for children (14.5 vs. 12.9%E), teenagers (15.5 vs. 13.9%E) and adults (18.0 vs. 16.4%E) with no difference in protein intake between high and non/low consumer groups for older adults or WCBA. The MDI of total fat was higher among high consumers compared to non/low consumers for children (34.5 vs. 33.1%E); however, there was no difference in total fat intake between consumer groups for any other population group examined. There was no difference in the MDI of saturated fat, MUFA or PUFA between consumer groups for any population group. High consumers of ‘fresh beef and lamb’ had a higher MDI of carbohydrate compared to non/low consumers for teenagers (47.6 vs. 50.1%E), adults (40.6 vs. 44.3%E) and WCBA (41.8 vs. 44.6%E) with no difference in carbohydrate intake between consumer groups for children or older adults. The MDI of total sugars and dietary fibre was lower among high consumers of ‘fresh beef and lamb’ compared to non/low consumers for adults (total sugars: 15.9 vs. 17.4%, dietary fibre: 21.4 vs. 24.2 g/10 MJ) while there was no difference in total sugar or dietary fibre intake between consumer groups for any other population group examined. There was no difference in the MDI of free sugars or salt between consumer groups for any population group examined. 

For micronutrients, the MDI of niacin was higher among high consumers compared to non/low consumers for children (42.2 vs. 39.0 mg/10 MJ) with no difference in niacin intake between consumer groups for any other population group examined (Table 3). The MDI of vitamin B6 was higher among high consumers compared to non/low consumers for WCBA (2.3 vs. 2.0 mg/10 MJ) with no difference in vitamin B6 intake between consumer groups for any other population group examined. The MDI of vitamin B12 was higher among high consumers compared to non/low consumers for children (6.8 vs. 5.7µg/10 MJ) and WCBA (4.4 vs. 2.9µg/10 MJ) with no difference in vitamin B12 intake between consumer groups for teenagers, adults or older adults. High consumers of ‘fresh beef and lamb’ had a higher MDI of zinc compared to low consumers in all population groups examined (children: 10.7 vs. 8.3 mg/10 MJ, teenagers: 11.3 vs. 9.7 mg/10 MJ, adults: 11.7 vs. 8.0 mg/10 MJ, older adults: 10.9 vs. 7.4 mg/10 MJ, WCBA: 9.4 vs. 6.8 mg/10 MJ). The MDI of potassium was higher among high consumers compared to non/low consumers for children (3523 vs. 3034 mg/10 MJ) with no difference in potassium intake between consumer groups for any other population group examined. There was no difference in the MDI of vitamin A, vitamin D, vitamin K, folate or iron between high and non/low consumers of ‘fresh beef and lamb’ for any population group examined.

### 3.4. Markers of Nutrition and Health Status among Non/Low and High Consumers of ‘Fresh Beef and Lamb’ (for Adults Only) 

For all population groups examined (18–64 y, 65–90 y, WCBA), there were no differences observed in mean systolic or diastolic BP, cholesterol, lipoprotein or triglyceride values between high consumers and non/low consumers of ‘fresh beef and lamb’ or in the proportion of the population with values outside generally accepted cut-offs indicating high BP or cholesterol (Table 4). Furthermore, there were no differences in the mean values of biochemical markers of nutritional status for serum 25-hydroxyvitamin D, serum vitamin B12, serum ferritin, haemoglobin or urinary Na:K between high consumers and non/low consumers of ‘fresh beef and lamb’ in any population group examined (18–64 y, 65–90 y, WCBA) or in the proportion of the population with values outside generally accepted cut-offs indicating low/deficient status. 

## 4. Discussion

This study provides information on the role of ‘fresh beef and lamb’ in the diets of the Irish population (5–90 years) and its association with markers of nutrition and health status. This study found that a large proportion of people living in Ireland, including vulnerable groups such as the elderly and WCBA, consumed ‘fresh beef and lamb’ and that ‘fresh beef and lamb contributed to intakes of a number of important nutrients including protein, MUFA, vitamin D, niacin, vitamin B6, vitamin B12, iron and zinc. For nutrients for which excess may have potential adverse health effects such as total fat, saturated fat and salt, ‘fresh beef and lamb’ contributed relatively small proportions to overall intakes of these nutrients. While higher consumption of ‘fresh beef and lamb’ was associated with higher intakes of total fat and lower intakes of carbohydrate and dietary fibre in some age groups, it was also associated with higher intakes of protein, niacin, vitamin B6, vitamin B12, zinc and potassium and lower intakes of total sugars. Furthermore, in adults, higher consumption of ‘fresh beef and lamb’ was not associated with an increased risk of cardio-metabolic diseases (as indicated by measures of systolic and diastolic BP, cholesterol, lipoprotein and Na:K) or better nutritional status (as indicated by biochemical markers of vitamin D, B12 and iron status). 

In the current study, ‘fresh beef and lamb’ was consumed by 68–84% of the population aged 5–90 years in Ireland (children and teenagers: 84%, adults 18–64 y: 76%, adults 65 y+: 73%, WCBA: 68%) with ‘beef’ more commonly consumed (63–83% consumers) than ‘lamb’ (11–30% consumers). These findings are similar to those reported in national dietary surveys in Italy, where 80–84% of children and teenagers and 75% of adults (younger and older) consumed ‘beef and veal’ [63] and in the US, where 78% of adults aged 18–64 y consumed ‘fresh beef’ [22]. However, the proportion of consumers in our study is higher than those reported in the UK, where 60% of children and teenagers and 65–70% of adults consumed ‘beef, veal, lamb and dishes’ and in Australia, where 44% of children and teenagers and 47% of adults consumed ‘beef and lamb’ [20,24]. It is interesting to note that in Ireland, older adults (65–90 years) were the highest consumers of lamb (30% compared to <20% across all other age groups), which is similar to findings from our UK counterparts (23% consumers aged 65+y compared to 11–17% across other age groups) [24]. This is also in line with global reports that older adults consider red meat (e.g., beef and sheep meat) as an important staple in their diet and is a reflection of the traditional dietary pattern of this generation in Ireland [64,65]. 

The recent EAT-Lancet commission report recommended an extreme reduction in red and processed meat consumption as part of a healthy diet from sustainable food systems and suggested a daily intake of 0–28 g for ‘beef, lamb and pork’ [1]. This study found that the MDI of ‘beef and lamb’ in the Irish population aged 5–90 y ranged from 19 to 43 g/d and generally increased with increasing age (5–12 y: 19 g, 13–18 y: 33 g, 18–64 y: 43 g, 65–90 y: 41 g and WCBA: 36 g). Similar intakes were reported for population groups in the UK (27–60 g/d), Italy (38–51 g/d) and Australia (38–53 g/d) whereas lower intakes were reported in Switzerland (16–21 g/d) and the Netherlands (5–16 g/d) [19,20,24,63,66]. Current intakes (both in Ireland and other countries) lie above the recommended range from the EAT-Lancet commission (particularly for teenagers and adults) and would require a significant dietary shift to meet these recommendations [1]. In line with findings from studies in Australia and Switzerland, this study found that teenage boys and men aged 18–64 y had higher intakes of ‘beef and lamb’ (40.2 and 55.7 g/d, respectively) than girls/women of the same age (25.1 and 29.7 g/d, respectively) [20,21,66]. 

It is well documented that relative to its energy profile, the nutritional quality that red meat provides is often under-valued [25,67]. This study adds to this evidence showing that relative to its contribution to energy intake across age groups (4–10%), ‘fresh beef and lamb’ contributed greater proportions of protein (11–20%) and MUFA (9–16%), similar proportions of total fat (7–14%) and saturated fat (7–16%) and smaller proportions of carbohydrate (1–2%), total sugars (1–2%), free sugars (0.3–1%) and dietary fibre (2–3%). Similarly, in the Netherlands, the UK and Australia relative to their contribution to energy intake across age groups (1–10%), ‘beef and lamb’ contributed to greater proportions of protein (3–19%) and similar proportions of total and saturated fat (2–10%) across all ages [19,20,21,24]. The contribution of ‘fresh beef and lamb’ to protein intakes is particularly notable as nutrient rich-high quality protein foods such as red meats can play an important role in helping people meet their essential nutrient needs and is particularly important for vulnerable groups including older adults for healthy ageing [16,68,69].

Fresh red meat was also shown to be a key contributor to key micronutrients such as vitamin B12, iron and zinc [25,67,68] and a recent review has highlighted that nutrient claims for red meat could be made for four out of the seven nutrients of public health concern designated by the US Dietary Guidelines for Americans, including sodium, potassium, iron and vitamin D, nutrients which were also identified as nutrients of public health concern in European populations [18]. In this study, relative to energy intakes, fresh ‘beef and lamb’ contributed greater proportions of zinc (18–27%), vitamin B12 (15–30%), vitamin D (10–16%), niacin (9–18%) and iron (7–13%) and similar proportions of vitamin A (7–9%), vitamin B6 (6–12%), potassium (5–8%), vitamin K (4–6%) and total folate (3–4%) which is in line with findings from the Netherlands, the UK and Australia where relative to energy intake (1–10%) beef and lamb (and their dishes) contributed to greater proportions of vitamin B12 (5–13%), vitamin D (3–8%), vitamin A (4–7%), iron (8–14%) and zinc (4–21%) [19,20,21,24]. A recent study investigating the association between red meat intakes and micronutrient intakes of UK females from the NDNS found that those with red meat intakes <40 g/d were more likely to have micronutrients intakes below the LNRI for zinc, iron, vitamin B12 and potassium and lower vitamin D intakes than those with intakes >40 g/d [25]. The findings of this study and the available literature further highlight the important role of red meat for micronutrient intakes and may be particularly relevant for vulnerable population groups in light of the restrictive limits proposed by the EAT-Lancet commission. Furthermore, a recent modelling study has shown that while the partial replacement of red and processed meat with plant-based alternatives improves overall diet quality, it may adversely affect the intake of some micronutrients, especially zinc and vitamin B12, further highlighting the important role of red meat in the diet [70].

Previous research from the US and Australia has shown a positive contribution of beef consumption to essential macronutrient and micronutrient intakes, such as protein, zinc, iron, and B vitamins with groups who choose to consume leaner cuts of beef with the lowest fat content having higher intakes of protein as well as vitamins B3, B6, B12, iron, phosphorus, and zinc and lower intakes of total energy, fat and carbohydrates [21,22,23]. In this study, we found that those who had higher intakes of fresh ‘beef and lamb’ had higher intakes of protein (children, teens and adults), niacin (children), vitamin B6 (WCBA), vitamin B12 (children and WCBA), zinc (all ages) and potassium (children) and lower intakes of carbohydrate (teens, adults and WCBA) and total sugars (adults) but also higher total fat (children) and lower dietary fibre (adults). A recent study from the NDNS also found that with increasing red meat intake, intakes of protein, MUFA, niacin, B6, iron and zinc intakes were significantly higher (and proportion with intakes below the LNRI decreased for zinc and iron), intakes of carbohydrate and total sugars intake were lower; however, total and saturated fat intakes also increased [26]. This raises the point that it is important to consider the cut and cooking methods as these have been linked to the fat content in red meats and FBDG do advise consuming lean red meat for protein and micronutrient content [2,67].

This study also investigated the association of ‘fresh beef and lamb’ consumption with markers of nutrition and health status in adults and found that higher consumption of ‘fresh beef and lamb’ was not associated with increased risk factors of cardio-metabolic diseases (as indicated by mean values and proportions of the population with values outside generally accepted cut-offs indicating high BP, cholesterol or Na:K). This study also found that higher consumption of ‘fresh beef and lamb’ was not associated with better nutritional status (as indicated by mean values and proportions of the population with values outside generally accepted cut-offs indicating low/deficient status for vitamin D, B12 and iron). Similarly, a study investigating the association between red meat intakes and micronutrient status of UK females did not find any significant difference for blood biomarkers of micronutrient status between groups with varying levels of red meat intake [25]. Furthermore, a recent study investigating associations between meat intakes and markers of health and cardio-metabolic diseases among adults in the UK found that while higher processed red meat consumption was associated with negative outcomes (e.g., higher BMI, hip circumference, higher TC, LDL-C, Hb A1c and PP), these associations were not observed for higher red meat consumption, supporting the evidence for dietary guidance on the reduction in processed red meat consumption but no justification for red meat reduction [26]. It was also shown that adolescent females consuming lean red meat had lower LDL levels and were less likely to have LDL-C values below cut-offs or elevated LDL:HDL ratio compared to girls with lower intakes of lean red meat suggesting that lean red meat may be included in a healthy adolescent diet without unfavourable effects on lipid values [27].

While the effects of red meat consumption compared to processed meat are well documented, a recent study also found that levels of atherogenic lipids and lipoproteins did not differ following consumption of diets with red meat compared to similar amounts of white meat concluding that the trial did not support evidence for choosing white over red meat for reducing CVD risk based on lipid and lipoprotein effects [14]. Another recent RCT investigated the effect of incorporating lean beef into a healthy dietary pattern and found that the benefits of a healthy, low saturated fat, Mediterranean-style diet were not attenuated by the inclusion of smaller to moderate amounts of lean beef further supporting evidence that the negative outcomes previously linked to red meat consumption are associated with the wider dietary patterns and confounding factors (e.g., BMI) associated with consuming red meat [12,15].

Overall, the findings of this study add to the evidence base of the existing literature supporting the role of ‘fresh beef and lamb’ (or fresh red meat) as part of a healthy diet particularly with respect to micronutrient intakes/status and as a source of high-quality protein which is particularly important for vulnerable populations including older adults [69,71]. Current intakes of ‘fresh beef and lamb’ both in Ireland and further afield are well above the particularly restrictive limits suggested by the EAT-Lancet commission and so would require significant shifts in dietary patterns to meet these limits which may have a negative impact on the intakes and status of some micronutrients [1]. However, dietary advice should educate consumers with respect to choosing lean options and utilising appropriate cooking methods to address intakes of nutrients of concern such as total fat, saturated fat and salt. Furthermore, research is continually ongoing to explore the potential to improve the nutritional composition of meat in terms of fatty acid profiles and micronutrient contents which may further improve the quality of meats including ‘fresh beef and lamb’ further supporting their role in a healthy diet [68,72,73,74,75].

### Strengths and Limitations

The main strengths of this study include the nationally representative samples of the population aged 5–90 years included in this study and the detailed dietary intake data (including brand level detail and customised recipes). Furthermore, the disaggregation of meat from composite foods is an important attribute of the present study as previous analysis has shown that failure to disaggregate composite foods substantially overestimates meat intakes by approximately 40% which may have important implications for epidemiological studies and for food safety [52]. However, it is important to note that in light of changing dietary patterns and a shift towards a more plant-based diet that these data from nationally representative surveys in Ireland were collected a number of years ago and so patterns may have shifted. National dietary surveys of these population groups are currently being conducted in Ireland and future studies should investigate the trends in fresh beef and lamb consumption in these population groups.

## 5. Conclusions

In summary, this study has investigated the nutritional role of ‘fresh beef and lamb’ in the diet of the population aged 5–90 years in Ireland using data from three nationally representative dietary surveys. The findings of this study add to the evidence base of the existing literature supporting the role of ‘fresh beef and lamb’ (or fresh red meat) as part of a healthy diet, particularly with respect to micronutrient intakes. Overall, this study found that a large proportion of people living in Ireland, including vulnerable groups such as children, older adults and WCBA, consumed ‘fresh beef and lamb’ (68–84% consumers across age groups) and highlighted the important contribution of ‘fresh beef and lamb’ consumption to intakes of key nutrients such as protein, monounsaturated fat, vitamins D, B12, niacin, iron and zinc while contributing relatively small proportions of nutrients of public health concern including total fat, saturated fat and salt.

This study also found that (within certain age groups) those who had higher intakes of fresh ‘beef and lamb’ had higher intakes of protein, niacin, vitamin B6, vitamin B12, zinc and potassium (but also total fat) and lower intakes of carbohydrate and total sugars (but also dietary fibre). In adults, older adults and WCBA, higher consumption of ‘fresh beef and lamb’ was not associated with increased risk factors of cardio-metabolic diseases (as indicated by measures of high BP, cholesterol or Na:K) nor was it associated with better or poorer nutritional status for vitamin D, B12 or iron. These findings show the important role of ‘fresh beef and lamb’ in the diet and may be useful for policymakers incorporating health and sustainability in updated FBDG.

## Figures and Tables

**Table 1:** Proportion of consumers (%) and mean daily intakes (g/d) of ‘fresh beef and lamb’, ‘beef’ and ‘lamb’ for the total population aged 5–90 years, by age group and sex.

	5–12 Years (*n* 594)			13–17 Years (*n* 441)			18–64 Years (*n* 1274)			65–90 Years (*n* 226)			WCBA 18–50 Years (*n* 487)		
	Beef and Lamb	Beef	Lamb	Beef and Lamb	Beef	Lamb	Beef and Lamb	Beef	Lamb	Beef and Lamb	Beef	Lamb	Beef and Lamb	Beef	Lamb
**All**															
Mean (g)	19.2 ^a^	16.6 ^a^	2.6 ^a^	32.8 ^b,c^	28.3 ^b,c^	4.5 ^a,b,c^	42.7 ^d^	36.3 ^d^	6.4 ^b,d^	40.8 ^b,d,e^	28.2 ^b,d,e^	12.6 ^e^	27.0 ^c^	23.7 ^c,e^	3.3 ^a,c^
Mean (g/10 MJ)	0.028 ^a^	0.024 ^a^	0.004 ^a^	0.039 ^b^	0.034 ^b^	0.005 ^a,b^	0.050 ^c^	0.043 ^c^	0.008 ^b,c^	0.058 ^c,d^	0.040 ^b,c,d^	0.018 ^d^	0.038 ^b,e^	0.033 ^b,e^	0.004 ^a,b^
SD (g)	18.6	16.7	6.7	32.1	29.6	11.9	42.6	40.0	17.2	39.7	33.7	23.7	28.8	28.0	10.8
Median (g)	14.7	13.2	0.0	25.1	21.4	0.0	33.5	26.7	0.0	32.5	20.1	0.0	20.3	16.0	0.0
IQR (g)	6.1–27.3	3.7–24.6	0.0–0.0	11.5–45.0	8.9–38.8	0.0–0.0	5.4–63.6	0.0–56.3	0.0–0.0	0.0–61.6	0.0–46.0	0.0–22.3	0.0–82.4	0.0–80.6	0.0–29.7
P97.5 (g)	64.4	55.7	21.6	111	103	40.7	151	136	61.5	149	114	73.7	100	100	37.5
Consumers (%)	84.2	78.8	18.9	84.4	83.0	19.5	76.0	70.2	16.2	72.6	59.3	30.1	67.8	62.8	10.9
**Boys/men**															
Mean (g)	20.3	17.7	2.6	40.2	34.9	5.2	55.7	47.1	8.6	44.7	31.9	12.8	-	-	-
Mean (g/10 MJ)	0.028	0.024	0.003	0.043	0.037	0.005	0.058	0.049	0.009	0.056	0.040	0.016			
SD (g)	20.6	17.8	7.6	36.5	34.8	12.3	48.6	46.1	21.0	40.2	34.4	24.5	-	-	-
Median (g)	14.6	13.6	0.0	31.6	26.7	0.0	46.9	37.2	0.0	39.5	25.4	0.0	-	-	-
IQR (g)	6.1–29.3	3.6–26.3	0.0–0.0	15.5–58.7	12.0–46.8	0.0–0.0	19.4–79.6	5.7–70.1	0.0–0.0	0.0–66.6	0.0–51.7	0.0–26.3			
P97.5 (g)	68.6	61.9	23.6	129	119	47.8	176	166	74.2	157	114	69.1			
Consumers (%)	83.2	77.7	17.9	88.8	87.9	21.0	82.2	75.7	18.6	73.6	62.3	29.2	-	-	-
**Girls/women**															
Mean (g)	18.1	15.6	2.5	25.1 *	21.4 *	3.7	29.7 *	25.6 *	4.1 *	37.3	25.0	12.4	-	-	-
Mean (g/10 MJ)	0.027	0.023	0.004	0.035	0.030	0.005	0.042 *	0.037 *	0.006	0.059	0.039	0.020			
SD (g)	16.4	15.5	5.8	24.6	21.0	11.4	30.6	29.1	12.0	39.2	32.9	23.1	-	-	-
Median (g)	14.8	12.9	0.0	19.1	16.4	0.0	23.2	17.5	0.0	27.1	13.3	0.0			
IQR (g)	6.1–25.4	3.8–23.6	0.0–0.0	7.1–38.3	5.6–31.4	0.0–0.0	0.0–46.9	0.0–41.4	0.0–0.0	0.0–58.1	0.0–41.6	0.0–19.8	-	-	-
P97.5 (g)	56.5	51.3	20.3	83.6	77.2	39.4	106	103	41.5	145	145	79.8			
Consumers (%)	85.1	79.9	19.8	79.7	77.9	18.0	69.8	64.7	13.8	71.7	56.7	30.8	-	-	-

Abbreviations: WCBA, women of childbearing age; g, gram; d, day; SD, Standard Deviation; IQR, Interquartile Range, P97.5; 97.5th percentile of intake. Statistical differences (*p* < 0.001) across age groups denoted by different superscript lowercase letters. * Statistically different (*p* < 0.001) from that of boys/men within the columns via independent samples *t*-tests and adjusted for multiple testing, differences between sexes are also highlighted in bold font.

**Table 2:** Contribution (%) of ‘fresh beef and lamb’ to mean daily intakes of energy and selected nutrients from all sources (including nutritional supplements) in consumers only aged 5–90 years, by age group.

	% Contribution				
	5–12 Years (*n* 500)	13–17 Years (*n* 372)	18–64 Years (*n* 968)	65–90 Years (*n* 164)	WCBA 18–50 Years (*n* 330)
Energy	4.7	6.5	8.2	9.5	7.3
Protein	11.5	15.2	18.8	20.1	16.9
Total fat	7.0	9.2	11.5	13.8	10.0
Saturated fat	6.9	9.6	13.0	15.7	11.3
MUFA	8.7	10.7	13.2	15.5	11.4
PUFA	4.0	4.8	5.4	7.6	4.7
Carbohydrate	1.5	2.0	2.3	2.4	2.4
Total sugars	1.0	1.5	1.8	1.7	1.8
Free sugars	0.3	0.7	1.0	0.6	0.9
Dietary fibre	2.0	2.9	3.1	3.1	3.0
Salt	5.9	7.1	8.6	8.0	8.6
Vitamin A	7.4	7.4	7.6	9.6	7.4
Vitamin D	12.4	16.2	14.0	10.1	13.1
Vitamin K	4.2	5.5	6.0	4.8	5.4
Niacin	9.4	12.5	15.0	18.0	13.5
Vitamin B6	6.4	8.8	10.6	12.2	9.7
Vitamin B12	15.0	21.9	29.9	29.5	27.8
Total folate	3.0	4.2	4.3	3.9	4.0
Iron	7.1	10.1	12.3	12.9	10.5
Zinc	18.1	23.4	27.4	27.3	24.6
Potassium	6.1	8.3	5.4	5.7	4.9

Abbreviations: WCBA, women of childbearing age; MUFA: Monounsaturated Fatty Acids; PUFA: Polyunsaturated Fatty Acids, Note: The contribution to intake of energy and nutrients (from all sources, i.e., including nutritional supplements) was estimated including the non-meat components of composite dishes.

**Table 3:** Mean daily intakes of energy and selected nutrients (energy adjusted) from food sources only (excluding nutritional supplements) in non/low and high consumers of ‘fresh beef and lamb’ in the total population aged 5–90 years, by age group.

	5–12 Years		13–17 Years		18–64 Years		65–90 Years		WCBA (18–50 Years)	
	Non/Low Consumers	High Consumers	Non/Low Consumers	High Consumers	Non/Low Consumers	High Consumers	Non/Low Consumers	High Consumers	Non/Low Consumers	High Consumers
	(*n* 197)	(*n* 198)	(*n* 146)	(*n* 147)	(*n* 423)	(*n* 424)	(*n* 75)	(*n* 75)	(*n* 162)	(*n* 162)
Fresh beef and lamb (g/d)	**3.3**	38.8 *	**6.2**	66.0 *	**4.9**	87.7 *	**2.5**	86.4 *	**0.3**	60.8 *
Consumers (%)	47.7	100	52.7	100	27.7	100	17.0	100	3.0	100
**Mean daily nutrient intakes (food sources only)**										
Energy (kcal)	1636	1734	**1861**	2158 *	**1919**	2106 *	1624	1778	**1634**	1843 *
Protein (%TE)	**12.9**	14.5 *	**13.9**	15.5 *	**16.4**	18.0 *	17.3	18.6	16.4	16.9
Total fat (%TE)	**33.1**	34.5 *	35.2	36.4	33.2	34.5	33.8	35.6	33.6	35.3
Saturated fat (%TE)	13.9	14.5	14.0	14.9	12.7	13.7	13.3	15.1	12.9	13.9
MUFA (%TE)	10.6	11.5	12.5	13.1	12.0	12.7	11.8	12.5	12.2	12.9
PUFA (%TE)	4.7	4.7	5.9	5.6	6.3	5.8	6.3	5.5	6.4	6.3
Carbohydrate (%TE)	53.5	50.6	**50.1**	47.6 *	**44.3**	40.6 *	44.5	42.2	**44.6**	41.8 *
Total sugars (%TE)	24.6	23.1	21.1	20.0	**17.4**	15.9 *	18.2	16.6	17.5	16.9
Free sugars (%TE)	17.2	15.5	14.4	13.9	9.0	8.5	7.9	7.5	9.1	9.2
Dietary fibre (g/10 MJ)	11.4	11.6	18.9	18.4	**24.2**	21.4 *	27.0	24.9	24.5	21.9
Salt (g/10 MJ)	72.7	74.9	76.7	75.6	61.8	64.9	51.4	56.0	52.9	57.4
Vitamin A (µg/10 MJ)	871	1089	914	1023	877	1093	1099	1410	837	993
Vitamin D (µg/10 MJ)	3.7	3.7	3.7	3.6	3.8	3.9	4.2	4.7	3.1	3.3
Vitamin K (µg/10 MJ)	130	135	134	137	178	161	201	166	177	172
Niacin (mg/10 MJ)	**39.0**	42.2 *	27.7	26.8	39.3	45.4	33.1	36.0	33.0	37.1
Vitamin B6 (mg/10 MJ)	2.8	2.9	3.8	3.3	2.5	2.9	2.3	2.7	**2.0**	2.3 *
Vitamin B12 (µg/10 MJ)	**5.7**	6.8 *	5.9	6.4	3.6	5.8	4.0	6.2	**2.9**	4.4 *
Total folate (µg/10 MJ)	318	318	346	315	311	320	293	343	251	267
Iron (mg/10 MJ)	13.0	13.8	16.8	13.9	11.8	12.9	10.1	11.4	10.4	11.2
Zinc (mg/10 MJ)	**8.3**	10.7 *	**9.7**	11.3 *	**8.0**	11.7 *	**7.4**	10.9*	**6.8**	9.4 *
Potassium (mg/10 MJ)	**3034**	3523 *	3209	3242	3650	3674	4031	3970	3699	3640

Abbreviations: WCBA, women of childbearing age; g, gram; d, day; kcal, kilocalories; %TE, percent total energy; MUFA, monounsaturated fatty acids; PUFA, polyunsaturated fatty acids; µg, microgram; mg, milligram; MJ, megajoule; * Statistically different (*p* < 0.001) from that of non/low consumers (stratified by sex and age group) within the rows via independent samples *t*-tests or Mann–Whitney U tests and adjusted for multiple testing, differences between consumer groups are also highlighted in bold font.

**Table 4:** Mean blood pressure, lipoprotein, cholesterol and biochemical markers of nutritional status values and the proportion (%) of the population with values outside generally accepted cut-offs indicating high or low/deficient status in non/low and high consumers of ‘fresh beef and lamb’ in adults aged 18–90 years, by age group.

	18–64 Years				65–90 Years				WCBA 18–50 Years			
	Non/Low Consumers		High Consumers		Non/Low Consumers		High Consumers		Non/Low Consumers		High Consumers	
	*n*	Mean	*n*	Mean	*n*	Mean	*n*	Mean	*n*	Mean	*n*	Mean
Systolic BP (mmHg)	376	123	369	122	63	139	59	140	144	112	145	113
Diastolic BP (mmHg)	376	77.5	369	77.8	63	81.4	59	81.4	144	75.0	145	75.3
Serum total cholesterol (mmol/L)	330	5.0	324	4.9	42	5.0	51	5.0	121	4.9	123	4.8
Serum triglycerides (mmol/L)	330	1.3	324	1.3	42	1.4	51	1.3	121	1.1	123	1.0
Serum direct HDL cholesterol (mmol/L)	327	1.5	321	1.6	41	1.7	51	1.7	120	1.7	123	1.7
Calculated LDL cholesterol (mmol/L)	323	2.8	316	2.8	41	2.7	51	2.8	119	2.7	123	2.7
Serum 25-hydroxyvitamin D (nmol/L)	274	57.6	267	60.7	29	53.5	34	50.3	101	56.1	105	60.4
Serum vitamin B12 (pmol/L)	289	300	266	317	35	294	44	316	103	284	100	286
Serum ferritin (ng/mL)	306	108	283	126	35	160	41	153	113	56.7	103	53.7
Haemoglobin (g/dL)	300	14.3	278	14.4	37	14.1	42	13.9	112	13.3	102	13.4
Urinary sodium (mmol/L)	322	95.1	317	102	42	83.0	54	88.0	123	91.6	126	105
Urinary potassium (mmol/L)	318	45.6	317	46.7	44	36.6	54	39.0	121	42.7	126	45.4
Urinary Na:K ratio	317	2.0	315	2.1	42	2.0	54	2.1	121	2.1	125	2.4
	***n***	**%**	***n***	**%**	***n***	**%**	***n***	**%**	***n***	**%**	***n***	**%**
Systolic BP ≥ 140 mmHg [54]	376	12.5	369	13.8	63	44.4	59	54.2	144	1.4	145	4.8
Diastolic BP ≥ 9 0 mmHg [54]	376	11.4	369	13.6	63	19.0	59	20.3	144	6.3	145	8.3
Serum total cholesterol > 5.2 mmol/L [55]	330	37.6	324	34.0	42	40.5	51	43.1	121	34.7	123	26.8
Serum 25-hydroxyvitamin D < 50 nmol/L [56]	274	43.1	267	40.1	29	51.7	34	50.0	101	47.5	105	44.8
Serum vitamin B12 < 148 pmol/L [57,58]	289	2.8	266	1.9	35	5.7	44	0.0	103	3.9	100	3.0
Serum ferritin < 15µg/L [3,59,60]	306	7.5	283	3.5	35	0.0	49	2.4	113	15.0	103	8.7
Haemoglobin < 13 g/dL (men) and <12 g/dL (women) [3]	300	4.3	278	3.6	37	8.1	42	9.5	112	5.4	102	7.8
Urinary Na:K ratio > 1 [61]	317	70.7	315	76.2	42	73.8	54	75.9	121	73.6	125	79.2

Abbreviations: mm, millimetre; Hg, mercury; L, litre; mmol, millimole; nmol, nanomole, pmol, picomole; g, gram; dL, decilitre; ng, nanogram; ml, millilitre, Na, sodium; K, potassium, No statistical differences (*p* < 0.001) were found between non/low and high consumers (stratified by sex and age group) for any blood pressure, lipoprotein, cholesterol or nutritional status values via independent samples t-tests, Mann–Whitney U tests or Chi-square tests and adjusted for multiple testing.

## Data Availability

The data presented in this study are available from the corresponding author upon reasonable request.

## References

[1] Willett W., Rockström J., Loken B., Springmann M., Lang T., Vermeulen S., Garnett T., Tilman D., DeClerck F., Wood A. (2019). Food in the Anthropocene: The EAT Lancet Commission on healthy diets from sustainable food systems. Lancet.

[2] Cocking C., Walton J., Kehoe L., Cashman K.D., Flynn A. (2020). The role of meat in the European diet: Current state of knowledge on dietary recommendations, intakes and contribution to energy and nutrient intakes and status. Nutr. Res. Rev..

[3] Scientific Advisory Committee on Nutrition (2010). Iron and Health.

[4] World Cancer Reserach Fund, American Institute for Cancer Research (2018). Diet, Nutrition, Physical Activity and Cancer: A Global Perspective. Continuous Update Project Expert Report 2018.

[5] Micha R., Wallace S.K., Mozaffarian D. (2010). Red and processed meat consumption and risk of incident coronary heart disease, stroke, and diabetes mellitus: A systematic review and meta-analysis. Circulation.

[6] Micha R., Michas G., Mozaffarian D. (2012). Unprocessed red and processed meats and risk of coronary artery disease and type 2 diabetes—An updated review of the evidence. Curr. Atheroscler. Rep..

[7] Pan A., Sun Q., Bernstein A.M., Manson J.E., Willett W.C., Hu F.B. (2013). Changes in red meat consumption and subsequent risk of type 2 diabetes mellitus: Three cohorts of US men and women. JAMA Intern. Med..

[8] Lippi G., Mattiuzzi C., Cervellin G. (2016). Meat consumption and cancer risk: A critical review of published meta-analyses. Crit. Rev. Oncol. Hematol..

[9] Wu J., Zeng R., Huang J., Li X., Zhang J., Ho J.C., Zheng Y. (2016). Dietary Protein Sources and Incidence of Breast Cancer: A Dose-Response Meta-Analysis of Prospective Studies. Nutrients.

[10] van den Brandt P.A. (2019). Red meat, processed meat, and other dietary protein sources and risk of overall and cause-specific mortality in The Netherlands Cohort Study. Eur. J. Epidemiol..

[11] Leroy F., Cofnas N. (2020). Should dietary guidelines recommend low red meat intake?. Crit. Rev. Food Sci. Nutr..

[12] Papier K., Fensom G.K., Knuppel A., Appleby P.N., Tong T.Y.N., Schmidt J.A., Travis R.C., Key T.J., Perez-Cornago A. (2021). Meat consumption and risk of 25 common conditions: Outcome-wide analyses in 475,000 men and women in the UK Biobank study. BMC Med..

[13] Stanton A.V., Leroy F., Elliott C., Mann N., Wall P., De Smet S. (2022). 36-fold higher estimate of deaths attributable to red meat intake in GBD 2019: Is this reliable?. Lancet.

[14] Bergeron N., Chiu S., Williams P.T., King S.M., Krauss R.M. (2019). Effects of red meat, white meat, and nonmeat protein sources on atherogenic lipoprotein measures in the context of low compared with high saturated fat intake: A randomized controlled trial. Am. J. Clin. Nutr..

[15] Fleming J.A., Kris-Etherton P.M., Petersen K.S., Baer D.J. (2021). Effect of varying quantities of lean beef as part of a Mediterranean-style dietary pattern on lipids and lipoproteins: A randomized crossover controlled feeding trial. Am. J. Clin. Nutr..

[16] Binnie M.A., Barlow K., Johnson V., Harrison C. (2014). Red meats: Time for a paradigm shift in dietary advice. Meat Sci..

[17] Wyness L. (2016). The role of red meat in the diet: Nutrition and health benefits. Proc. Nutr. Soc..

[18] Cashman K.D., Hayes A. (2017). Red meat’s role in addressing ‘nutrients of public health concern’. Meat Sci..

[19] van Rossum C.T.M., Buurma-Rethans E.J.M., Vennemann F.B.C., Brants H.A.M., de Boer E.J., Ocké M.C. (2020). The Diet of the Dutch. Results of the First Two Years of the Dutch National Food Consumption Survey 2012–2016.

[20] Sui Z., Raubenheimer D., Rangan A. (2017). Consumption patterns of meat, poultry, and fish after disaggregation of mixed dishes: Secondary analysis of the Australian National Nutrition and Physical Activity Survey 2011–12. BMC Nutr..

[21] Bowen J., Baird D., Syrette J., Noakes M., Baghurst K. (2012). Consumption of beef/veal/lamb in Australian children: Intake, nutrient contribution and comparison with other meat, poultry and fish categories. Nutr. Diet..

[22] An R., Nickols-Richardson S., Alston R., Shen S., Clarke C. (2019). Total, Fresh, Lean, and Fresh Lean Beef Consumption in Relation to Nutrient Intakes and Diet Quality among U.S. Adults, 2005–2016. Nutrients.

[23] O’Neil C.E., Zanovec M., Keast D.R., Fulgoni V.L., Nicklas T.A. (2011). Nutrient contribution of total and lean beef in diets of US children and adolescents: National Health and Nutrition Examination Survey 1999–2004. Meat Sci..

[24] Bates B., Lennox A., Prentice A., Bates C., Page P., Nicholson S., Swan G. (2014). National Diet and Nutrition Survey: Results from Years 1–4 (Combined) of the Rolling Programme (2008/2009–2011/2012).

[25] Derbyshire E. (2017). Associations between Red Meat Intakes and the Micronutrient Intake and Status of UK Females: A Secondary Analysis of the UK National Diet and Nutrition Survey. Nutrients.

[26] Hobbs-Grimmer D.A., Givens D.I., Lovegrove J.A. (2021). Associations between red meat, processed red meat and total red and processed red meat consumption, nutritional adequacy and markers of health and cardio-metabolic diseases in British adults: A cross-sectional analysis using data from UK National Diet and Nutrition Survey. Eur. J. Nutr..

[27] Bradlee M.L., Singer M.R., Moore L.L. (2014). Lean red meat consumption and lipid profiles in adolescent girls. J. Hum. Nutr..

[28] European Commission (2020). FOOD 2030 Pathways for Action. Healthy, Sustainable and Personalised Nutrition.

[29] Central Statistics Office (2007). Census 2006 Principal Demographic Results.

[30] Central Statistics Office (2002). Census 2002 Principal Demographic Results.

[31] Nelson M., Atkinson M., Meyer J. (1997). A Photographic Atlas of Food Portion Sizes.

[32] Ministry of Agriculture Fisheries and Food (1997). Food Portion Sizes.

[33] Food Standards Agency (2002). McCance and Widdowson’s the Composition of Foods.

[34] Holland B., Welch A., Unwin I.D., Buss D.H., Paul A.A., Southgate D.A.T. (1995). McCance and Widdowson’s the Composition of Foods.

[35] Holland B., Widdowson E.M., Unwin I.D., McCance R.A., Buss D.H. (1988). Cereal and Cereal Products: Third Supplement to McCance and Widdowson’s the Composition of Foods.

[36] Holland B., Unwin I.D., McCance R.A., Buss D.H. (1989). Milk Products and Eggs: Fourth Supplement to McCance and Widdowson’s the Composition of Foods.

[37] Holland B., Widdowson E.M., Unwin I.D., Buss D.H. (1991). Vegetables, Herbs and Spices: Fifth Supplement to McCance and Widdowson’s the Composition of Foods.

[38] Holland B., Unwin I.D., Buss D.H. (1992). Fruit and Nuts: First Supplement to the Fifth Edition of McCance and Widdowson’s the Composition of Foods.

[39] Holland B., Brown J., Buss D.H. (1993). Fish and Fish Products: Third Supplement to the Fifth Edition of McCance and Widdowson’s the Composition of Foods.

[40] Chan W., Brown J., Church S.M., Buss D.H. (1994). Miscellaneous Foods. Fourth Supplement to McCance & Widdowson’s the Composition of Foods.

[41] Chan W., Brown J., Church S.M., Buss D.H. (1995). Meat Poultry and Game. Fifth Supplement to McCance & Widdowson’s the Composition of Foods.

[42] Chan W., Brown J., Church S.M., Buss D.H. (1996). Meat Products and Dishes. Sixth Supplement to McCance & Widdowson’s the Composition of Foods.

[43] Holland B., Welch A., Buss D.H. (1996). Vegetable Dishes: Second Supplement to the Fifth Edition of McCance and Widdowson’s the Composition of Foods.

[44] Black L.J., Walton J., Flynn A., Cashman K.D., Kiely M. (2015). Small Increments in Vitamin D Intake by Irish Adults over a Decade Show That Strategic Initiatives to Fortify the Food Supply Are Needed. J. Nutr..

[45] Li K., McNulty B.A., Tiernery A.M., Devlin N.F.C., Joyce T., Leite J.C., Flynn A., Walton J., Brennan L., Gibney M.J. (2016). Dietary fat intakes in Irish adults in 2011: How much has changed in 10 years?. Br. J. Nutr..

[46] Walton J., Kehoe L., McNulty B.A., Nugent A.P., Flynn A. (2017). Intakes and sources of dietary sugars in a representative sample of Irish adults (18–90 y). Proc. Nutr. Soc..

[47] Morrissey E., Giltinan M., Kehoe L., Nugent A.P., McNulty B.A., Flynn A., Walton J. (2020). Sodium and Potassium Intakes and Their Ratio in Adults (18–90 y): Findings from the Irish National Adult Nutrition Survey. Nutrients.

[48] Cashman K.D., Muldowney S., McNulty B., Nugent A., FitzGerald A.P., Kiely M., Walton J., Gibney M.J., Flynn A. (2013). Vitamin D status of Irish adults: Findings from the National Adult Nutrition Survey. Br. J. Nutr..

[49] Hopkins S.M., Gibney M.J., Nugent A.P., McNulty H., Molloy A.M., Scott J.M., Flynn A., Strain J., Ward M., Walton J. (2015). Impact of voluntary fortification and supplement use on dietary intakes and biomarker status of folate and vitamin B-12 in Irish adults. Am. J. Clin. Nutr..

[50] Moore Heslin A., O’Donnell A., Buffini M., Nugent A.P., Walton J., Flynn A., McNulty B.A. (2021). Risk of Iron Overload in Obesity and Implications in Metabolic Health. Nutrients.

[51] Iwahori T., Ueshima H., Torii S., Saito Y., Kondo K., Tanaka-Mizuno S., Arima H., Miura K. (2017). Diurnal variation of urinary sodium-to-potassium ratio in free-living Japanese individuals. Hypertens. Res..

[52] Cosgrove M., Flynn A., Kiely M. (2007). Impact of disaggregation of composite foods on estimates of intakes of meat and meat products in Irish adults. Public Health Nutr..

[53] Krebs-Smith S.M., Kott P.S., Guenther P.M. (1989). Mean proportion and population proportion: Two answers to the same question?. J. Am. Diet. Assoc..

[54] World Health Organization (2013). A Global Brief on Hypertension Silent Killer, Global Public Health Crisis.

[55] British Cardiac Society, British Hyperlipidaemia Association, British Hypertension Society, British Diabetic Association (1998). Joint British recommendations on prevention of coronary heart disease in clinical practice. Heart.

[56] Institute of Medicine (2011). Dietary Reference Intakes for Calcium and Vitamin D.

[57] EFSA Panel on Dietetic Products Nutrition and Allergies (2015). Scientific Opinion on Dietary Reference Values for cobalamin (vitamin B12). EFSA J..

[58] Devalia V., Hamilton M.S., Molloy A.M., British Committee for Standards in Haematology (2014). Guidelines for the diagnosis and treatment of cobalamin and folate disorders. Br. J. Haematol..

[59] World Health Organization (2007). Assessing the Iron Status of Populations.

[60] World Health Organization (2011). Serum Ferritin Concentrations for the Assessment of Iron Status and Iron Deficiency in Populations. Vitamin and Mineral Nutrition Information System.

[61] World Health Organization (2012). Sodium Intake for Adults and Children.

[62] Fagerland M.W. (2012). T-tests, non-parametric tests, and large studies—A paradox of statistical practice?. BMC Med. Res. Methodol..

[63] Leclercq C., Arcella D., Piccinelli R., Sette S., Le Donne C. (2009). The Italian National Food Consumption Survey INRAN-SCAI 2005–06: Main results in terms of food consumption. Public Health Nutr..

[64] Holman B.W.B., Fowler S.M., Hopkins D.L. (2020). Red meat (beef and sheep) products for an ageing population: A review. Int. J. Food Sci..

[65] Murphy D. (2021). Exploring evidence of lost and forgotten Irish food traditions in Irish cookbooks 1980–2015. Folk Life.

[66] Tschanz L., Kaelin I., Wróbel A., Rohrmann S., Sych J. (2022). Characterisation of meat consumption across socio-demographic, lifestyle and anthropometric groups in Switzerland: Results from the National Nutrition Survey menuCH. Public Health Nutr..

[67] McAfee A.J., McSorley E.M., Cuskelly G.J., Moss B.W., Wallace J.M., Bonham M.P., Fearon A.M. (2010). Red meat consumption: An overview of the risks and benefits. Meat Sci..

[68] De Smet S., Vossen E. (2016). Meat: The balance between nutrition and health. A review. Meat Sci..

[69] Richi E.B., Baumer B., Conrad B., Darioli R., Schmid A., Keller U. (2015). Health Risks Associated with Meat Consumption: A Review of Epidemiological Studies. Int. J. Vitam. Nutr. Res..

[70] Vatanparast H., Islam N., Shafiee M., Ramdath D.D. (2020). Increasing Plant-Based Meat Alternatives and Decreasing Red and Processed Meat in the Diet Differentially Affect the Diet Quality and Nutrient Intakes of Canadians. Nutrients.

[71] Kouvari M., Tyrovolas S., Panagiotakos D.B. (2016). Red meat consumption and healthy ageing: A review. Maturitas.

[72] Daley C.A., Abbott A., Doyle P.S., Nader G.A., Larson S. (2010). A review of fatty acid profiles and antioxidant content in grass-fed and grain-fed beef. Nutr. J..

[73] Scollan N.D., Price E.M., Morgan S.A., Huws S.A., Shingfield K.J. (2017). Can we improve the nutritional quality of meat?. Proc. Nutr. Soc..

[74] Meale S.J., Chaves A.V., He M.L., McAllister T.A. (2014). Dose-response of supplementing marine algae (*Schizochytrium* spp.) on production performance, fatty acid profiles, and wool parameters of growing lambs. J. Anim. Sci..

[75] Duffy S.K., O’Doherty J.V., Rajauria G., Clarke L.C., Cashman K.D., Hayes A., O’Grady M.N., Kerry J.P., Kelly A.K. (2017). Cholecalciferol supplementation of heifer diets increases beef vitamin D concentration and improves beef tenderness. Meat Sci..

